# Increase in chemokines CXCL10 and CCL2 in blood from pigs infected with high compared to low virulence African swine fever virus isolates

**DOI:** 10.1186/1297-9716-44-87

**Published:** 2013-10-01

**Authors:** Emma Fishbourne, Evelyne Hutet, Charles Abrams, Roland Cariolet, Marie-Frédérique Le Potier, Haru-H Takamatsu, Linda K Dixon

**Affiliations:** 1 The Pirbright Institute, Pirbright, Woking, Surrey GU24 0NF, UK; 2 Anses, Unité Virologie Immunologie, Zoopôle Les Croix, 22440 Ploufragan, France; 3 Université européenne de Bretagne, 5 Boulevard Laennec, 35000 Rennes, France

## Abstract

Modulation of the expression of chemokines and chemokine receptors in whole blood was compared following infection of pigs with high and low virulence isolates of African swine fever virus. Levels of mRNAs for CCL2, CCL3L1, CCL4, CXCL10, CCR1 and CCR5 were significantly increased in at least one time point following infection in two experiments and CCL5, CCR9 and CXCR4 mRNA were significantly increased in one of the experiments. The results showed that greatest fold increases in mRNAs for CXCL10 and CCL2 were observed following infection of pigs. CXCL10 mRNA was increased by up to 15 fold in infected compared to uninfected pigs. CXCL10 protein was also detected in serum from pigs infected with the high virulence Benin 97/1 isolate. Levels of CCL2 mRNA were increased in pigs infected with high virulence Benin 97/1 isolate compared to low virulence OURT88/3 isolate and this correlated with an increase of greater than 30 fold in levels of CCL2 protein detected in serum from pigs infected with this isolate. An increase in overall chemotaxis active compounds in defibrinated plasma samples from Benin 97/1 infected pigs was observed at 3 days post-infection (dpi) and a decrease by 7 dpi as measured by chemotaxis assay using normal pig leucocytes *in vitro*. Increased levels of CXCL10 may either contribute to the activation of lymphocyte priming toward the Th1 phenotype or induction of T lymphocyte apoptosis. Increased levels of CCL2, a chemoattractant for macrophages, may result in increased recruitment of monocytes from bone marrow thus increasing the pool of cells susceptible to infection.

## Introduction

African swine fever virus (ASFV) is a large double-stranded DNA virus and is the only member of the *Asfarviridae* family. The genome varies between 170 and 193 kbp and encodes 150 to 167 open reading frames. The virus has been established in a sylvatic cycle in East and southern Africa, involving warthogs and soft ticks of *Ornithodoros* species, for an extended period. In these hosts infection is inapparent and the virus can cause persistent infections. However, most ASFV isolates cause an acute haemorrhagic fever, African swine fever (ASF), in domestic pigs and wild boar resulting in high mortality. ASFV is endemic or causes sporadic outbreaks of disease in most sub-Saharan countries in Africa [1,2]. Outside Africa ASF is currently endemic in Sardinia, the Trans Caucasus region and Russian Federation [3].

Most isolates of ASFV are highly virulent in domestic pigs and can result in mortality approaching 100%. Some less virulent isolates have been described. Moderately virulent isolates result in reduced mortality of 30% to 50% and low virulence isolates cause few clinical signs and very low mortality. The virus replicates primarily in the cytoplasm of cells of the monocyte macrophage lineage, although other cells have been shown to be infected at later stages of disease. Acute ASF disease is characterised by high levels of viremia, thrombocytopenia, leukopenia, damage to vascular endothelial cells and induction of disseminated intravascular coagulation [4]. Apoptosis of bystander non-infected lymphocytes in tissues and blood is also a characteristic of the infection [5,6]. In lymph tissues apoptosis is observed around infected macrophages, suggesting that factors released from these cells are involved in induction of apoptosis [7]. Infection with low virulence virus strains including OURT88/3 or NHV/P68 can induce an immune response that protects pigs from challenge with related virulent viruses [8,9]. Transient low levels of viremia can be detected, as well as low levels of virus in some lymph tissues in pigs infected with these viruses. The protection induced by OURT88/3 isolate is dependent on CD8+ T cells and is abrogated by depletion of this cell subset [10].

Complete genome sequences of low and high virulence ASFV isolates have been determined. The low virulence OURT88/3 isolate lacks an 8 kbp sequence near the left genome end which encodes 5 copies of multigene family (MGF) 360 and two copies of MGF 530 [11]. Deletion of orthologs of these genes, plus one additional copy of MGF 360 from a high virulence isolate, was shown previously to result in increased type I interferon production and stimulation of interferon response genes [12]. The OURT88/3 genome also has interruptions in the EP402R, EP153R and MGF360 18R genes [11]. EP402R encodes a transmembrane protein, CD2v, that is required for binding of virus-infected cells and extracellular virus particles to red blood cells and may have a role in impairing lymphocyte activation [13,14]. The EP153R ORF encodes a C-type lectin protein that has been implicated in inhibiting up-regulation in cell surface expression of MHC Class I and also inhibiting apoptosis in infected cells [15,16]. A number of other ASFV genes have been identified which have roles in evading host defences. These include the A238L protein which inhibits transcriptional activation of host immune response genes dependent on several transcription factors. These factors interact with the N-terminal domain of the p300/CBP transcriptional co-activator and include NF-κB, NFAT and c-Jun [17-19]. The I329L protein has been shown to inhibit signalling through Toll-like receptors 3 and 4 [20].

Chemokines play important roles in regulating immune responses and other processes. Many viruses have developed strategies to manipulate the chemokine response to modulate the outcome of infections. For example both poxviruses and herpesviruses use strategies including virus-encoded secreted proteins which bind specific chemokines, virus encoded mimics of chemokines and chemokine receptors [21]. ASFV-encoded proteins which modulate gene transcription in infected macrophages may also be involved in altering chemokine responses.

In this study we compared chemokine responses in pigs infected with high virulence and low virulence ASFV isolates by using quantitative reverse transcriptase PCR (qRTPCR) to measure changes, following infection, in levels of mRNAs in whole blood for selected chemokines and chemokine receptors. These mRNAs were selected for study because they are expressed in macrophages, the main target cells for ASFV replication. We also measured serum levels of CCL2, CXCL8 and IFN γ and total chemotactic substances. The mRNA level for CXCL10 was up-regulated by the greatest fold in infected compared to uninfected pigs and was higher in pigs infected with the high virulence isolate Benin 97/1 compared to low virulence OURT88/3 isolate. CCL2 mRNA levels were also greatly increased in pigs infected with the high virulence Benin 97/1 isolate. This correlated with the high levels of CCL2 detected by ELISA in sera from pigs infected with the virulent isolates Benin 97/1 and Uganda 1965. The results suggest that in pigs infected with the virulent isolates, elevation in levels of CCL2 may be a mechanism by which monocytes are recruited to circulation increasing the pool of cells susceptible to infection.

## Materials and methods

### Virus strains and cells

The African swine fever virus strains used OURT88/3, Benin 97/1, Uganda 1965, have been described previously [8,11,22,23]. These were isolated from tissue or blood from infected pigs (Benin 97/1, Uganda 1965) or from an infected tick (OURT88/3). Virus isolates OURT88/3 and Benin 97/1 are genotype I and Uganda 1965 is genotype X as defined by partial sequencing of the B646L gene encoding the major capsid protein p72 [24]. Viruses were passaged a minimum number of times in primary pig macrophages. The adherent cell population from pig bone marrow was cultured in Earle’s saline including 10% porcine serum, 200 U/mL penicillin and 200 μg/mL streptomycin. The dose for pig infection was 10^4^ HAD_50_ or TCID_50_ of each strain.

### RNA extraction

Whole blood was collected from pigs in PAXgene Blood tubes (Qiagen, Limberg Netherlands) and stored at -20 °C. RNA was prepared from the thawed samples using the PAXgene Blood RNA extraction kit (Qiagen) according to the manufacturer’s instructions. The quantity and quality of RNA extracted was assessed using a spectrophotometer (Nanodrop ND-1000, Nanodrop technologies, Wilmington, USA) to measure the ratio of absorbance at 260 and 280 nm and Agilent RNA 6000 Nano kit (Agilent Technologies,Santa Clara, USA) and Agilent 2100 bioanalyser to measure the RNA integrity number (RIN).

### Quantitative reverse transcriptase PCR

First strand cDNA was produced from RNA using the Superscript III First Strand Synthesis System for RT-PCR (Invitrogen, Life Technologies, Carlsbad, USA) according to the manufacturer’s instructions. Primers used for cDNA synthesis were a mixture of random hexamers. Quantitative PCR was carried out using gene specific primers for CCL2, CCL3L1, CCL4, CCL5, CCR1, CCR5, CCR7, CCR9, CXCL2, CXCL8, CXCL10, CXCR3L and CXCR4 as described previously [25] and in the legends to the relevant figures and the SYBR Green JumpStart TaqReadyMix (Sigma-Aldrich, St Louis, USA). Amplification conditions were as described previously [25] and included 2 min 94 °C, followed by 40 cycles of 15 s at 94 °C and 1 min at 56 to 63 °C. An MX3005P real time PCR machine (Stratagene, Agilent Technologies) was used for amplification. Control reactions were carried out using samples prepared without a reverse transcriptase step.

### ELISA assays

ELISA assays to detect Interferon gamma, CXCL8 and CCL2 in defibrinated plasma samples were carried out according to the manufacturer’s recommendations (Kingfisher Biotech. St Paul, USA). Plasma samples were diluted to 30% in reagent diluents before use in assays.

### Western blotting

Protein from defibrinated plasma samples was separated on 15% SDS/PAGE gels and transferred to Hybond-LFP (PVDF) membrane (GE Healthcare, Little Chalfont, UK). Membranes were incubated with polyclonal antibody against porcine CXCL10 (Kingfisher Biotech) followed by anti-rabbit antibody conjugated to horse radish peroxidase and bound antibodies were detected by enhanced chemiluminescence.

### Chemotaxis assays

Plasma was separated from whole blood in EDTA by centrifugation at 600 *g* for 10 min. Plasma was defribinated by treatment with 25 mM CaCl_2_ at 37 °C for 1 h, followed by centrifugation at 600 *g* for 5 min to remove debris and storage at -80 °C. Virus was removed from infected samples by using a centrifugation spin column with 300 000 kDa molecular weight cut-off (Vivaspin, Sartorius, Goettingen, Germany). Samples were diluted to 30% in DMEM plus Hepes medium before use in chemotaxis assays.

Porcine peripheral blood leucocytes (PBLs) from uninfected healthy donor pigs were prepared by collection of pig blood in heparin, mixing with an equal volume of 6% dextran and incubation at 37 °C for 30 min. The supernatant was removed and PBLs pelleted at 450 *g* for 4 min. Cells were washed in calcium and magnesium free PBS and any remaining red blood cells were lysed using 150 mM ammonium chloride for 5 min on ice. PBL migration to chemoattractants was measured in a 96 well chemotaxis chamber (ChemoTx, NeuroProbe, Gaithersburg, MD, USA). Isolated porcine PBLs were re-suspended at a final concentration of 5 × 10^6^ cells per mL in DMEM plus Hepes. The wells in the chamber were filled with 29 μL of defibrinated plasma samples from infected or uninfected pigs in DMEM plus Hepes media. Media only was used as negative control and recombinant CXCL8 (R & D Systems, Minneapolis, USA) diluted in PBS with 0.1% BSA to give a concentration of 500, 100, 10 and 1 ng mL^-1^ was used as a positive control. A poly-carbonate cell permeable membrane (5 μm pore size) was placed on top of the wells and 25 μL of the PBL suspension pipetted on to the filters. The plates were incubated for 2-2 ½ h at 37 °C in 5% CO2. Non-migrating cells were removed from the top of the filter by aspiration. After incubation the cells were aspirated from the upper surface of the membrane and 15 μL of 2 mM EDTA was added to each filter. The plate was then incubated for a further 30 min at 4 °C before removing the EDTA by gentle wiping of the surface of the membrane with a tissue and then washing with PBS. The plate was then centrifuged at 400 *g* for 10 min before carefully removing the filter. The PBLs that had migrated into the wells were read by an ELISA plate reader at 450 nm following incubation for 4 h with Cell Counting Kit-8 (CCK-8, Dojindo Molecular Technologies, Inc, Rockville, USA) or immediately by a MACSQuant® Analyser (Miltenyibiotec, Bergisch Gladbach, Germany).

### White blood cell count

Lymphocyte counts were obtained from blood with a MS9 hematology analyzer (Melet Schloesing laboratoires, Osny, France).

### Statistical tests

The variation measured for a treated group was analyzed using a general linear model. Data obtained were compared to the control group (statistical significance is indicated in the figures) or to another treated group. Statistical groupings were obtained using Tukey’s method with 95% confidence. Statistical tests on blood cell counts were performed using Systat 9 software (Systat Software Inc., Point Richmond, USA), with a limit of significance of *p* < 0.05.

### Animal welfare

Experiments at Ploufragan were performed according to the animal welfare experimentation agreement given by the Direction des Services Vétérinaires des Côtes d’Armor (AFSSA registration number B-22-745-1), under the responsibility of Marie-Frédérique Le Potier (agreement number 22-17).

## Results

### Infections of pigs with ASFV

To investigate the modulation of the host chemokine response caused by ASFV, pigs were infected with ASFV isolates of low (OURT88/3) or high virulence (Benin 97/1 or Uganda 1965) and whole blood samples were collected at different days post-infection (dpi). Samples from two experiments were analysed. In experiment 1 one group of 6 pigs was infected with the low virulence genotype I OURT88/3 isolate, a second group of 5 pigs was infected with genotype I high virulence Benin 97/1 isolate and a third group of 4 pigs was infected with genotype X high virulence Uganda 1965 isolate. In this experiment the pigs infected with OURT88/3 isolate were 6 weeks younger than the other pigs. In experiment 2 one group of 6 pigs was infected with OURT88/3 isolate and a second group of 6 pigs was infected with Benin 97/1 isolate. These pigs were age-matched. The clinical signs and levels of viremia detected have already been reported for experiments 1 and 2 [26]. As expected all of the pigs infected with high virulence isolates, Benin 97/1 and Uganda 65 developed clinical and post-mortem signs typical of acute ASF and were euthanized between 5 and 7 dpi. High levels of viremia were detected, by qPCR, from 3 dpi. Mean levels of viremia at 3 dpi in pigs infected with Benin 97/1 were 7.5 log10 HAD_50_/mL and remained at this level at 5 dpi. In pigs infected with Uganda (1965) isolate viremia at day 3 was lower (mean value 2.5 log_10_ HAD_50_/mL) but increased by day 5 to levels similar to those observed in Benin 97/1 infected pigs (mean value 6.7 log_10_ HAD_50_/mL). The pigs infected with OURT88/3 isolate had few if any clinical signs. In experiment 1, 4 pigs infected with OURT88/3 developed a transient low fever of 4 to 5 days between 10 and 16 days post-immunisation and transient low viremia of log 10^1-2^ genome copies per mL was detected in 1 pig. In experiment 2, 3 pigs infected with OURT88/3 developed a transient low fever, two of these had fever between 12 and 18 dpi and the third between 6 and 10 and 12 and 18 days. Low and transient viremia of log 10 ^1-2^ genome copies per mL was detected in 2 pigs.

### Changes in levels of porcine chemokine and chemokine receptor mRNAs in whole blood following infection of pigs with low and high virulence ASFV isolates

**Figure 1:**
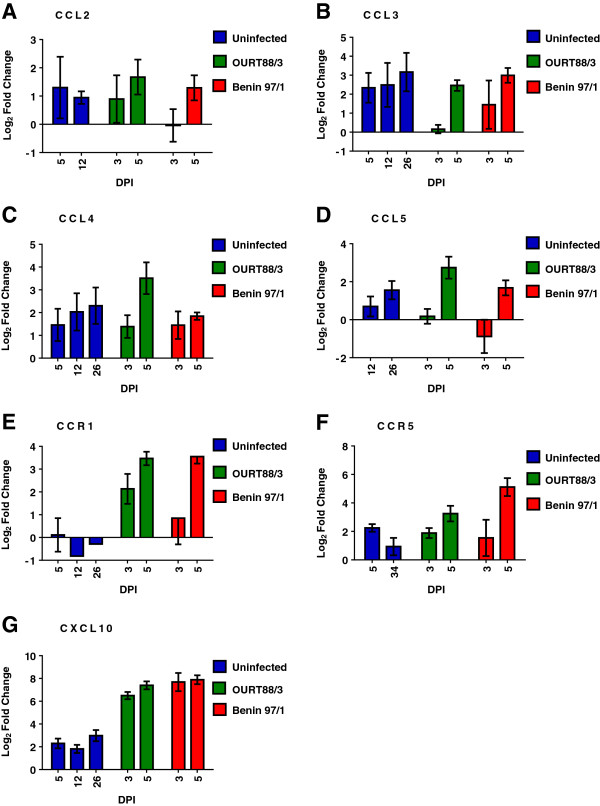
**Changes in levels of mRNAs for porcine CC ligands following infection of pigs with different ASFV isolates.** Changes in mRNA for chemokine and chemokine receptor genes in porcine blood cells were estimated by real time RT PCR and are shown for samples from experiment 1. Pigs were either uninfected or infected with ASFV isolates, OURT88/3 or Benin97/1. Samples were taken 3, 5, 12 or 26 dpi and RNA was extracted. mRNA levels were measured by real time RT-PCR using SYBR Green (Sigma). Changes are shown as Log_2_ fold changes on the y axis compared to 1 time point pre-infection to give the calibrator sample. Data from real time RT PCR for **(A)** CCL2, **(B)** CCL3L, **(C)** CCL4, **(D)** CCL5, **(E)** CCR1, **(F)** CCR5, **(G)** CXCL10 are shown. The dpi samples were collected is shown on the x axis and the different isolates are indicated on the right of each panel. The mean for each group ± SEM is shown, *n* = 3-6 pigs.

**Figure 2:**
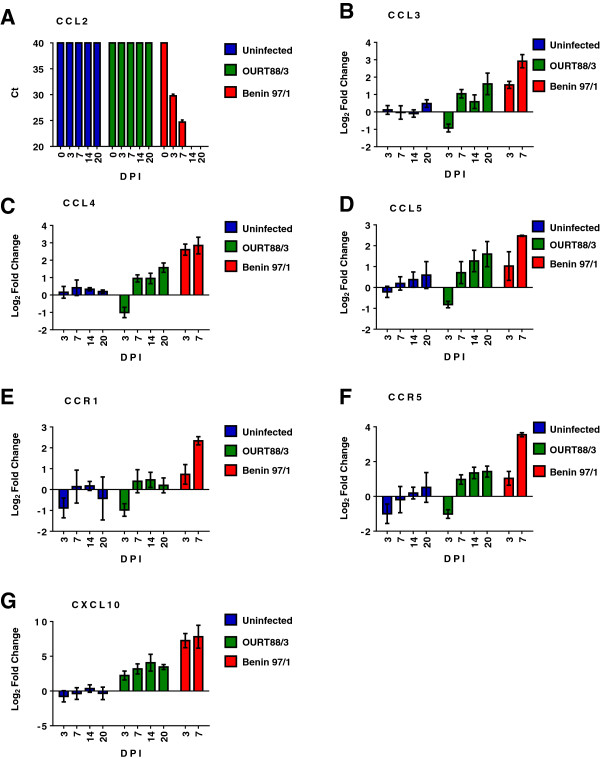
**Changes in levels of mRNAs for porcine CC ligands following infection of pigs with different ASFV isolates.** Changes in mRNA for chemokine and chemokine receptor genes in porcine blood cells were estimated by real time RT PCR and are shown for samples from experiment 2. Pigs were either uninfected or infected with ASFV isolates, OURT88/3 or Benin97/1. Samples were taken at 0 3, 7, 14 or 20 dpi and RNA was extracted. mRNA levels were measured by real time RT-PCR using SYBR Green (Sigma). Changes are shown as Log_2_ fold changes (y-axis) compared to 2 time points pre-infection to give the calibrator sample. No amplification was detected for CCL2 mRNA in calibrator samples so data shown is the Ct value. Data from real time RT PCR for **(A)** CCL2, **(B)** CCL3L, **(C)** CCL4, **(D)** CCL5, **(E)** CCR1, **(F)** CCR5, **(G)** CXCL10, are shown. The dpi is shown on the x axis and the different isolates are indicated on the right of each panel. The mean for each group ± SEM is shown, *n* = 3-6 pigs.

Chemokine and chemokine receptor genes selected for analysis were inflammatory chemokines and chemokine receptors known to be expressed in macrophages. Changes in mRNA levels were measured relative to calibrator samples collected from pigs pre-infection. Additional file 1 shows mean changes in mRNA levels in infected compared to uninfected pigs and indicates statistically different groupings determined using Tukey’s method with 95% confidence. Figures 1 and 2 show graphs indicating the changes in mRNA levels detected in experiments 1 and 2 respectively. Those chemokine and chemokine receptor mRNAs shown to be significantly increased in samples from infected compared to uninfected pigs included in both experiments CXCL10, CCL2, CCL3L1, CCL4, CCR1 and CCR5. In addition mRNAs for CCR9 and CXCR4 were significantly altered in experiment 2 and in experiment 1 CCL5 was altered.

### Comparison of changes in mRNAs for chemokine and chemokine receptor genes in whole blood from pigs infected with low compared to high virulence isolates

In experiment 1, comparison between samples from Benin 97/1 and OURT88/3 infected pigs and uninfected pigs showed a significant increase (*P* = 0.001) in mRNA level for CXCL10 at 3 and 5 dpi compared to uninfected pigs. The mean fold changes compared to uninfected pigs were log_2_ 7.7 and 7.9 for samples from the Benin 97/1 infected pigs and 6.5 and 7.4 for samples from the OURT88/3 infected pigs, at 3 and 5 dpi respectively. A significant decrease (*P* = 0.004) in mRNA level of CCL3L1 at 3 dpi and an increase (log_2_ 3.5 or 3.6) for CCR1 at 5 dpi was also observed in pigs infected with Benin 97/1 and OURT88/3 isolates (*P* = 0.001). Levels of CCL5 mRNA were significantly increased by log_2_ 2.7 fold (*P* = 0.002) in samples collected from pigs infected with OURT88/3 at 5 dpi compared to those from pigs infected with either OURT88/3 or Benin 97/1 at 3 dpi (Additional file 1 and Figure 1).

In samples from infected pigs mRNA levels for CCL2 and CCL4 were up-regulated compared to control samples from uninfected pigs although no significant difference was observed in these mRNA levels when samples from pigs infected with Benin 97/1 and OURT88/3 isolates were compared. Levels of mRNA for CCR1 and CCR5 were increased significantly (*P* = 0.001 or 0.010 respectively) at 5 dpi in samples from Benin 97/1 infected pigs compared to uninfected pigs. Additional file 1 shows the mean fold change for each mRNA analysed.

**Figure 3:**
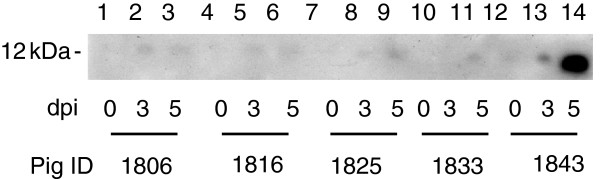
**Western blot to detect CXCL10 in plasma samples from ASFV infected and uninfected pigs.** Levels of CXCL10 protein were measured in de-fibrinated plasma from pigs uninfected or infected with ASFV isolates Benin 97/1. Samples were separated by SDS-PAGE and Western blotting followed by immunodetection using a polyclonal antibody against porcine CXCL10 followed by a secondary antibody couple to HRP. Bound antibodies were detected by enhanced chemiluminesence. The results from samples collected from individual pigs (numbered below) at different dpi are shown. The position of a 12 kDa molecular weight marker run in parallel is indicated.

In experiment 2 significant differences in mRNA levels were seen between samples for CCL3, CCL4, CXCL2, CXCL10, CCR1, CCR5 and CXCR4 as grouped using the Tukey method with 95% confidence (Additional file 1 and Figure 3). Levels of mRNA for CXCL10 were significantly increased (*P* = 0.000) in samples from Benin 97/1 and OURT88/3 infected pigs at all of the time points compared to uninfected pigs. mRNA levels for CXCL10 were significantly higher (*P* = 0.000) in samples from the Benin 97/1 infected pigs compared to OURT88/3 infected pigs, except for at 14 dpi with OURT88/3. Mean fold changes compared to samples from uninfected pigs were greater than log_2_ 7 at days 3 and 7 from pigs infected with Benin 97/1 and log_2_ 2.2 at 3 dpi with OURT88/3 rising to a maximum of log_2_ 4.1 at 14 dpi.

For CCL2 no Ct value was recorded for samples from uninfected pigs or the OURT88/3 infected pigs at any of the time points. There was a large increase in mRNA level in samples from the Benin 97/1 infected pigs as Ct values were recorded at 3 and 7 dpi indicating an increase in expression of equivalent to 10 to 15 Ct. CCL3L1 mRNA levels were significantly increased in samples from Benin 97/1 infected pigs collected at 7 dpi compared to all other samples except the samples from 3 dpi with Benin 97/1 and 20 dpi with OURT88/3. The CCL3L1 mRNA levels were significantly (*P* = 0.000) higher (log_2_ 2.9) in samples at 3 dpi with Benin 97/1 compared to in samples from uninfected pigs or from OURT88/3 infected pigs at 3 dpi (log_2_ -0.9). In the samples from pigs infected with OURT88/3 at 20 dpi levels of CCL31 mRNA had increased to log_2_ 1.6 compared to earlier time points. CCL4 mRNA levels were significantly (*P* = 0.000) higher in samples (up to log_2_ 2.8 fold increase) from the Benin 97/1 infected pigs at both 3 and 7 dpi compared to samples from the uninfected pigs and the OURT88/3 infected pigs except at 20 dpi when levels of mRNA increased by log_2_ 1.6 fold in samples from OURT88/3 infected pigs. There was a significant decrease (*P* = 0.000) in mRNA levels of CCL4 in samples from the OURT88/3 infected pigs at 3 dpi compared to all other samples.

Levels of mRNA for chemokine receptors CCR1 and CCR5 were significantly increased (*P* = 0.003 or 0.000 respectively) by log_2_ 2.3 or 3.5 in samples from the Benin 97/1 infected pigs at 7 dpi compared to samples from the uninfected pigs and OURT88/3 infected pigs at 3 dpi. In samples from Benin 97/1 infected pigs at 7 dpi compared to other samples, mRNA levels for CXCR4 were significantly increased (*P* = 0.004) by a small amount log_2_ 1.5. Additional file 1 shows the mean fold change in mRNA level for samples from each infected group in experiment 2 and results are presented graphically in Figure 2. The largest fold changes were observed for mRNAs for CCL2 and CXCL10. Up-regulation of mRNAs for both genes was greater in the pigs infected with the virulent Benin 97/1 ASFV isolate compared to the pigs infected with the low virulence OUR T88/3 isolate.

### Comparison of protein levels of Interferon γ, CXCL8, CCL2 and CXCL10 in plasma samples from ASFV infected compared to uninfected pigs

**Figure 4:**
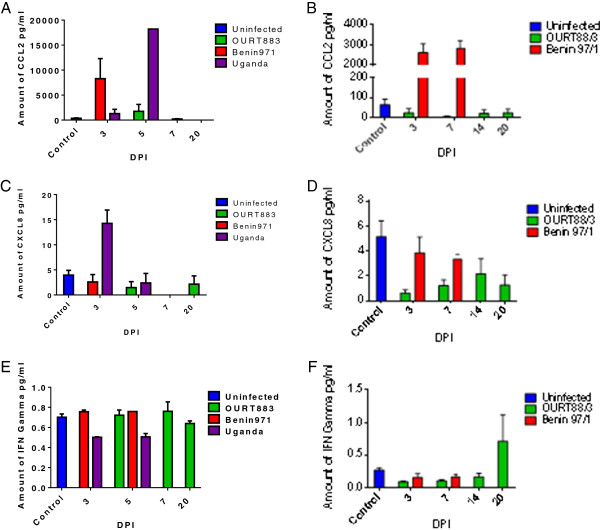
**Amount of CCL2, IFN γ and CXCL8 in plasma samples from ASFV infected and uninfected pigs.** Pigs were infected with high virulence ASFV isolates Benin 97/1, Uganda 1965 or low virulence isolate OURT88/3 and plasma samples were collected at different days post-infection as indicated on the x-axis. The amount of CCL2 **(**Panels **A** and **B)**, CXCL8 **(**Panels **D** and **E)** and IFN γ **(**Panels **E** and **F)** present was estimated by ELISA assay. Values for samples from pigs infected with different isolates or uninfected pigs are indicated by different coloured bars. Error bars indicate SEMs. The results from experiment 1 are shown in panels **A**, **C**, **E** and from experiment 2 in **B**, **D**, **F**.

To determine if increased mRNA levels for CCL2 and CXCL10 may be correlated with an increase in levels of the proteins in plasma from infected compared to uninfected pigs, we analysed levels of CCL2 by ELISA and CXCL10 by Western blotting. In parallel levels of CXCL8 and IFNγ were compared as controls. Figure 4 shows the amounts in pg/ml of CCL2, CXCL8 and IFNγ in samples from experiments 1 and 2 respectively. In samples from experiment 1 (Figure 4a) the level of CCL2 was significantly increased (*P* = 0.001) at 3 dpi in samples from the Benin 97/1 infected pigs compared to the other groups. Greatly increased levels of CCL2 were also detected in samples collected at 5 dpi with Uganda 1965 isolate. There were very low levels of IFN γ and CXCL8 and no significant difference was identified between samples (Figure 4c and e).

In samples from experiment 2 the level of CCL2 was significantly increased at 3 and 7 dpi with the Benin 97/1 isolate compared to the other groups (*P* = 0.000) (Figure 4b). Levels of CCL2 in the Benin 97/1 infected pigs were approximately 40 times higher than those in other groups. In contrast very low levels of IFN γ and CXCL8 were detected in all of the samples (Figure 4d, f). At 3 dpi there was a significant decrease (*P* = 0.032) in the plasma levels of CXCL8 in the samples from the OURT88/3 infected pigs compared to other samples. No significant difference in IFNγ levels was detected between samples from different groups.

De-fibrinated filtered plasma were separated by SDS-PAGE and blotted onto PVDF membranes. CXCL10 was detected by Western blotting. A positive control consisted of samples from porcine PBLs stimulated with porcine IFNγ (not shown). Bands of the correct size (~12 kDa) were only detected in the samples from the pigs infected with the Benin 97/1 isolate at 3 and 5 dpi in experiment 1 (Figure 3) and at 7 dpi in experiment 2 (data not shown). The signal detected was very weak except in samples from one pig (Figure 3 pig 1843). In this pig low levels of CXCL10 were detected in the sample pre-infection increasing in amount dramatically by 5 dpi.

The increase in levels of CCL2 detected in pigs infected with Benin 97/1 correlates with increases in levels of mRNA for CCL2 and suggests the white blood cell fraction may be the source of CCL2. The low levels of CXCL10 detected by Western blotting in samples from Benin 97/1 infected pigs also suggest some correlation with changes in mRNA levels detected.

### Measurement of chemotactic substances in plasma from ASFV infected pigs

**Figure 5:**
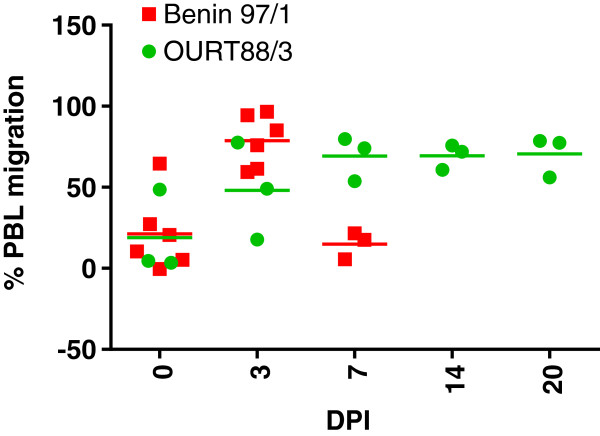
**Migration of cells from uninfected pigs to plasma samples from infected pigs.** Pigs were infected with high virulence ASFV isolate Benin 97/1 or low virulence OURT88/3 and at different dpi plasma samples were collected. Migration of peripheral blood leucocytes from uninfected donor samples to a 30% dilution of defibrinated plasma samples from uninfected or infected pigs was measured using the Neuroprobe 96 well chemotaxis assay. The number of PBLs that migrated is represented by the chemotaxis index (y axis). The x axis indicates the day post-infection samples were collected. Square red shapes indicate data from individual pigs infected with Benin 97/1 and green circles those from OURT88/3 infected pigs. Each shape represents data from 1 pig and is the mean of 3 or 4 replicates.

To determine if changes in levels of chemokines, including CCL2, result in changes in total chemotactic substances, migration of PBLs from three different uninfected donor pigs across a membrane in a transwell was assessed in response to defibrinated plasma from ASFV-infected or uninfected pigs. The results for samples from experiment 2 are shown on Figure 5. The migration of the PBLs is represented by the chemotactic index shown on the y-axis. PBLs from all 3 donor pigs showed an increase in migration to samples from the Benin 97/1 infected pigs collected at 3 dpi and a decrease to samples collected at 7 dpi. PBLs from donor pigs 1 and 2 both showed an increase in migration to samples from the OURT88/3 infected pigs compared to the uninfected controls. Levels of migration of PBLs in response to samples from OURT88/3 infected pigs increased from 20 dpi compared to those from 3 dpi.

### Analysis of the white blood cell counts from ASFV infected pigs

**Figure 6:**
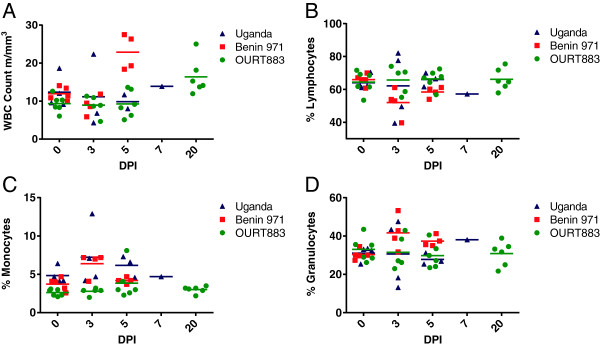
**White blood cell counts in different cell populations following infection of pigs with ASFV.** Pigs were infected with high virulence ASFV isolates Benin 97/1, Uganda 1965 or low virulence isolate OURT88/3. At different dpi, as indicated on the x-axis, blood samples were collected and the total white blood cell count **(**Panel **A)**, and proportions of lymphocytes **(**Panel **B)**, monocytes **(**Panel **C)** or granulocytes **(**Panel **D)** were measured in samples from different pigs. The values obtained for samples from individual Benin 97/1 infected pigs are shown in red, for OURT88/3 infected pigs in green and Uganda 1965 infected pigs in purple. Mean values are indicated by a horizontal bar.

We monitored changes in the white blood cell population during infection to identify changes which might be influenced by the chemotactic substances present. In experiment 1, a difference was recorded in the white blood cell count for pigs infected with Benin 97/1 and Uganda (1965) isolates compared to pigs pre-infection (Figure 6a). The white blood cell count decreased in pigs infected with the virulent Benin 97/1 isolate and Uganda (1965) isolates at 3 dpi and decreased further in the Uganda (1965) infected pigs at 5 dpi. However in blood from Benin 97/1 infected pigs at 5 dpi, cell count increased significantly to numbers above those present pre-infection (*p* = 0.001). The white blood cell count for the OURT88/3 infected pigs remained constant and increased slightly at 20 dpi (see Figure 6a).

The percentage of lymphocytes in white blood cells reduced in the Benin 97/1 and Uganda (1965) isolate infected pigs at 3 dpi, but this difference was not significant (see Figure 6b). In experiment 2 there was a significant decrease in the percentage of lymphocytes in the white blood cell population at 3 dpi with Benin 97/1 isolate (data not shown).

In the pigs infected with the virulent Benin 97/1 and Uganda 1965 isolates there was a significant increase in the percentage (*P* = 0.001) of monocytes at 3 dpi. This increased further at 5 dpi in pigs infected with Uganda 1965 isolate but had decreased at 5 dpi to pre-infection levels in pigs infected with Benin 97/1 isolate. The percentage of monocytes in the pigs infected with OURT88/3 isolate remained relatively constant until day 20 when a slight increase was observed (see Figure 6c).

In experiment 1 the percentage of granulocytes showed a trend to increase at 3 and 5 dpi in the Benin 97/1 infected pigs but these changes were not significant (Figure 6d). Thus significant changes in white blood cell counts and proportions of different sub-populations occurred following infection which may be influenced by and influence the profile of chemotactic substances and receptors.

## Discussion

The aim of this study was to compare chemokine and chemokine receptor gene expression in blood samples from pigs infected with high and low virulence isolates of ASFV to identify changes which occur. This would improve understanding of the differences in pathogenesis and immune responses induced by these isolates. Pigs infected with high virulence isolates Benin 97/1 and Uganda 1965 developed clinical signs by 3 or 4 dpi and were euthanized by day 5 or 7. High levels of viremia were detected in blood and tissues as expected. Of the 12 pigs in the 2 experiments infected with the low virulence isolate OURT88/3, 6 pigs developed transient low fever and low levels of viremia were detected in 4 pigs.

Part of the study involved measuring levels of mRNAs for chemokine and chemokine receptors in whole blood samples. This analysis focussed on chemokines and chemokine receptors produced in monocytes or macrophages, which are the target cells for ASFV replication. Some variation was observed in results between experiment 1 and 2 (see Additional file 1 and Figures 1 and 2). In experiment 2 more of the genes analysed had significantly different levels of mRNA in infected compared to uninfected pigs. Possibly this was because in experiment 2 pigs infected with the different isolates were the same age whereas in experiment 1 the pigs infected with virulent isolates were 6 weeks older than those infected with the low virulence isolate OURT88/3. Also in experiment 2 calibrator samples were collected at both one week and one day prior to infection whereas in experiment 1 calibrator samples were collected at one day before infection only. Those mRNAs for CCL2, CCL31, CCL4, CXCL10, CCR1 and CCR5 were significantly increased following infection in both experiments in at least one time-point and CCL5, CCR9 and CXCR4 mRNA were significantly altered in one of the experiments. In both experiments the levels of mRNA for chemokine CXCL10 were increased by the greatest fold in samples from infected pigs compared to uninfected pigs. In addition CXCL10 was also detected weakly by Western blotting in plasma samples in serum from several pigs infected with Benin 97/1 isolate. This correlation between changes in expression of mRNA in blood cells and protein levels in serum for the pigs infected with Benin 97/1 isolate indicates that blood cells may be the source of this chemokine in the circulation. CXCL10 is part of the group of interferon-stimulated genes and it plays an important role during different viral infections by induction of cell activation, chemotaxis and lymphocyte priming toward the Th1 phenotype. This may be important for mounting an effective immune response. Increased expression of CXCL10 has been reported in a number of virus infections including influenza, HIV, West Nile virus and Nipah virus [25,27-31]. However, CXCL10 has also been reported to have a pro-apoptotic role in several cell types and in combination with IL-2 and/or IFNα, has been reported to induce apoptosis in T lymphocytes. Apoptosis induced by CXCL10 was shown to be dependent on p38 MAPK and pro-survival signals relied on sustained activation of P13K and transient activation of Akt [32]. Induction of apoptosis of bystander non-infected lymphocytes is a hallmark of acute ASFV infection [5-7,33,34]. In agreement with previous studies the percentage of lymphocytes in the white blood cell population decreased significantly at 3 or 4 dpi with the virulent Benin 97/1 or Uganda 1965 isolates, possibly due to induction of apoptosis. Since CXCL10-induced activation via its ligand CXCR3 can also trigger pro-survival signals, two possible effects of increased CXCL10 expression can be proposed [32]. CXCL10, together with other co-stimulating cytokines, may participate in the activation of T lymphocytes, promote survival and expansion of certain lymphocyte subsets, and induce chemotaxis toward the infected tissues. Alternatively, CXCL10 might contribute to trigger apoptosis in other subsets of T lymphocytes lacking appropriate sets of specific co-stimulating signals for survival. These different outcomes dependent on CXCL10 and other stimuli may trigger the differing responses in pigs infected with high compared to low virulence isolates that is, induction of apoptosis in pigs infected with virulent ASFV or stimulation of a Th1 response in pigs infected with the low virulence isolate.

CCL2 is another chemokine shown here to be differentially regulated following ASFV infection. In both experiments 1 and 2 levels of CCL2 detected by ELISA in plasma from pigs infected with virulent isolates Benin 97/1 (experiments 1 and 2) and Uganda 1965 (experiment 1) were dramatically increased by greater than 30 fold to more than 2000 pg/mL compared to samples from OURT88/3 infected or uninfected pigs. This was observed from 3 dpi with Benin 97/1 and 5 dpi with Uganda 1965 isolates. The increase in protein levels for CCL2 was correlated with a dramatic increase in levels of mRNA for CCL2 in blood cells from Benin 97/1 infected pigs in experiment 2. In experiment 1 a less dramatic increase in mRNA levels for CCL2 was observed in samples from the Benin 97/1 infected pigs, possibly the timing of a peak in mRNA expression was missed in the sampling. This indicates that white blood cells could be the source of CCL2 detected in plasma. Macrophages are the main cells producing CCL2 and production is enhanced as monocytes mature into macrophages. Macrophages of intermediate to mature phenotype are the main target cells for ASFV replication [35] and thus infected macrophages in blood could be the source of CCL2 detected. However non-infected macrophages in blood may also produce the CCL2 detected. CCL2 attracts monocytes through interaction with its receptor CCR2. Inducing an increase in CCL2 in blood could represent a mechanism the virus uses to attract susceptible cells to areas of infection and increase viral dissemination. In our experiments an increase in the percentage of monocytes in white blood cell fractions was observed in pigs infected with the virulent Benin 97/1 and Uganda 1965 isolates at 3 dpi. Possibly this may result from recruitment of monocytes from the bone marrow into the blood circulation caused by high levels of CCL2. In pigs infected with Benin 97/1 isolate, the percentage of monocytes in the white blood cell fraction had decreased by 5 dpi, possibly due to cell death caused by virus replication. Interestingly, in another study infection of pigs with virulent ASFV strain Armenia was also shown to result in an increase in the percentage of monocytes and of immature white blood cells in blood, suggesting that activation of hematopoiesis and release of lymphocytes and monoblasts from the bone marrow occurred following infection [36]. Measurement of chemotactic substances in plasma samples from infected and uninfected pigs indicated a higher level in samples from pigs infected with Benin 97/1 isolate at 3 dpi compared to samples from uninfected pigs or pigs infected with OURT88/3. This may in part be due to increased levels of CCL2 and CXCL10 detected in these samples.

Levels of mRNA for several other chemokines or chemokine receptors increased but by a lesser fold in whole blood cells following infection of pigs with ASFV. These included CCL3, CCL4, CXCL2, CCR1 and CCR5, which were all upregulated by greater than log_2_ 2 fold in samples from pigs infected with the virulent Benin 97/1 isolate compared to OURT88/3 isolate and uninfected pigs. Thus the effect of infection on chemokine response is likely to be complex. CCL3 and CCL4 are pro-inflammatory chemokines and can directly promote development of IFNγ producing lymphocytes [37]. The induction of IFNγ producing lymphocytes was shown previously to correlate with protection induced by the OURT88/3 isolate [26]. CXCL2 acts as a chemoattractant for neutrophils and can also activate or attract basophils, eosinophils, monocytes and lymphocytes [38]. Ligands for CCR5 include CCL3, CCL4 and CCL5, and up-regulation of this receptor would be important for regulating response to those chemokines. Although reagents were not available to measure up-regulation at the protein level of these chemokines in response to infection, this would be interesting to determine.

In a previous study we compared the effect on expression of the same chemokine and chemokine receptor genes following infection of macrophages *in vitro* with Benin 97/1 and OURT88/3 isolates [25]. The results showed increased levels of mRNA for CXCL10 were induced by both isolates in comparison with mock-infected cells. Levels of CXCL10 mRNA induced by Benin 97/1 infection *in vitro* were lower than for OURT88/3, contrasting with the data from *in vivo* infections described here. Also contrasting with the results described here from the studies of *in vivo* infections, levels of mRNA for CCL3, CXCL2, CCR1 and CCR5 were down-regulated compared to mock-infected cells following infection *in vitro*. In addition mRNAs for CXCR3 and CXCR4 were down-regulated *in vitro*. In our analysis of samples from pigs infected in vivo we have not distinguished between expression in infected cells compared to uninfected cells and therefore differences between the *in vitro* and *in vivo* results might result due to analysis of mRNA in uninfected cells rather than infected *in vivo*. Alternatively they may result from the local cytokine environment in infected pig blood inducing an altered pattern of gene expression in infected cells.

In conclusion our data have indicated some key differences in the patterns of chemokine expression in pigs infected with high virulence compared to low virulence ASFV isolates which may help to explain key differences in the pathogenesis and immune response of pigs to these isolates.

## Competing interests

The authors declare they have no competing interests.

## Authors’ contributions

EF participated in the design of the study and writing the manuscript, carried out qRTPCR, ELISA and chemotaxis assays. CA contributed to the design of the study and preparation of the manuscript. EH carried out the analysis of cell counts and participated in animal studies. RC contributed to the design and animal experiments. M-FleP contributed to the design of the study, completion of animal experiments and preparation of the manuscript. H-HT contributed to the design of the study and results analysis, LD contributed to the design of the study, results analysis and preparation of the manuscript. All authors read and approved the final manuscript.

## Supplementary Material

Additional file 1 **Comparison of changes in mRNA levels for chemokine and chemokine receptor genes at different dpi of pigs with high virulence ASFV isolate Benin 97/1 and low virulence ASFV isolate OURT88/3.** Changes in mRNA levels of chemokine and chemokine receptor genes in porcine blood cells were estimated by real time RT PCR. Pigs were either uninfected or infected with different ASFV isolates, OURT88/3 or Benin97/1. In experiment 1 samples were taken 3 and 5 dpi and in experiment 2 at 3, 7, and in addition, for OURT88/3 infected pigs, at 14 and 20 dpi. mRNA levels for chemokine and chemokine receptor genes were quantified by real time RT-PCR. Changes in mRNA levels are shown as Log_2_ fold changes compared to samples collected pre infection to give the calibrator sample for each pig. A general linear model was used to analyse the data obtained for the levels of mRNA for different chemokine genes. A comparison was made between samples from pigs infected with the different isolates for each day post-infection. Means that do not share a letter are significantly different. Grouping information was derived using the Tukey method and 95% confidence. To take into account variation between pigs, all the uninfected samples at the different time points were used for comparison with samples from infected isolates. Click here for file
