# Supplementary material for: Increase in chemokines CXCL10 and CCL2 in blood from pigs infected with high compared to low virulence African swine fever virus isolates
Source: Vet Res. 2013 Oct 1;44(1):87. doi: 10.1186/1297-9716-44-87 (PMC3832245; doi:10.1186/1297-9716-44-87)
Supplement: Additional file 1 — Comparison of changes in mRNA levels for chemokine and chemokine receptor genes at different dpi of pigs with high virulence ASFV isolate Benin 97/1 and low virulence ASFV isolate OURT88/3. Changes in mRNA levels of chemokine and chemokine receptor genes in porcine blood cells were estimated by real time RT PCR. Pigs were either uninfected or infected with different ASFV isolates, OURT88/3 or Benin97/1. In experiment 1 samples were taken 3 and 5 dpi and in experiment 2 at 3, 7, and in addition, for OURT88/3 infected pigs, at 14 and 20 dpi. mRNA levels for chemokine and chemokine receptor genes were quantified by real time RT-PCR. Changes in mRNA levels are shown as Log2 fold changes compared to samples collected pre infection to give the calibrator sample for each pig. A general linear model was used to analyse the data obtained for the levels of mRNA for different chemokine genes. A comparison was made between samples from pigs infected with the different isolates for each day post-infection. Means that do not share a letter are significantly different. Grouping information was derived using the Tukey method and 95% confidence. To take into account variation between pigs, all the uninfected samples at the different time points were used for comparison with samples from infected isolates. [file 1297-9716-44-87-S1.docx]

| Experiment 1 | | | | | | | |
| --- | --- | --- | --- | --- | --- | --- | --- |
|  |  | None | OURT88/3 | | Benin 97/1 | | *P* value |
| CCL2 | 3 | A | 0 | A | 0 | A | 0.269 |
|  | 5 | A | 1.5 | A | 1.3 | A |  |
| CCL3L | 3 | A | 0.2 | B | 1.4 | AB | 0.004 |
|  | 5 | A | 2.5 | A | 3.0 | A |  |
| CCL4 | 3 | A | 1.4 | A | 1.4 | A | 0.059 |
|  | 5 | A | 3.9 | A | 1.8 | A |  |
| CCL5 | 3 | AB | 0.2 | B | -0.9 | B | 0.002 |
|  | 5 | AB | 2.7 | A | 1.7 | AB |  |
| CXCL10 | 3 | B | 6.5 | A | 7.7 | A | 0.001 |
|  | 5 | B | 7.4 | A | 7.9 | A |  |
| CCR1 | 3 | B | 2.1 | AB | 0.8 | AB | 0.001 |
|  | 5 | B | 3.5 | A | 3.6 | A |  |
| CCR5 | 3 | B | 1.9 | B | 1.5 | B | 0.010 |
|  | 5 | B | 3.3 | AB | 5.1 | A |  |
| Experiment 2 | | | | | | | |
| CCL2 | 3 |  | >40 Ct |  | >10 Ct  increase |  |  |
|  | 7 |  | >40 Ct |  | >15 Ct  increase |  |  |
|  | 14 |  | >40 Ct |  | - |  |  |
|  | 20 |  | >40 Ct |  | - |  |  |
| CCL31 | 3 | CD | -0.9 | D | 1.6 | AB | 0.000 |
|  | 7 |  | 1.0 | BC | 2.9 | A |  |
|  | 14 |  | 0.6 | BC | - |  |  |
|  | 20 |  | 1.6 | CD | - |  |  |
| CCL4 | 3 | C | -1.0 | D | 2.6 | A | 0.027 |
|  | 7 |  | 1.0 | BC | 2.8 | A |  |
|  | 14 |  | 0.6 | BC | - |  |  |
|  | 20 |  | 1.6 | AB | - |  |  |
| CCL5 | 3 | A | -0.8 | A | 0.6 | A | 0.002 |
|  | 7 |  | 0.7 | A | 2.0 | A |  |
|  | 14 |  | 1.3 | A | - |  |  |
|  | 20 |  | 1.6 | A | - |  |  |
| CXCL2 | 3 |  | -0.7 | B | 0.8 | B | 0.0000 |
|  | 7 |  | -0.2 | B | 4.3 | A |  |
| CXCL10 | 3 | C | 2.2 | BC | 7.3 | A | 0.000 |
|  | 7 |  | 3.2 | B | 7.8 | A |  |
|  | 14 |  | 4.1 | AB | - |  |  |
|  | 20 |  | 3.5 | B | - |  |  |
| CCR1 | 3 | B | -0.1 | B | 0.7 | AB | 0.003 |
|  | 7 |  | 0.4 | AB | 2.3 | A |  |
|  | 14 |  | 0.5 | AB | - |  |  |
|  | 20 |  | 0.2 | AB | - |  |  |
| CCR5 | 3 | BC | -1.0 | B | 1.0 | B | 0.000 |
|  | 7 |  | 1.0 | C | 3.5 | A |  |
|  | 14 |  | 1.3 | B | - |  |  |
|  | 20 |  | 1.4 | AB | - |  |  |
| CCR7 | 3 | A | -0.8 | A | -0.1 | A | 0.003 |
|  | 7 |  | -0.9 | A | 0.2 | A |  |
|  | 14 |  | 0.0 | A | - |  |  |
|  | 20 |  | 0.2 | A | - |  |  |
| CCR9 | 3 | B | -0.8 | B | 0.4 | AB | 0.004 |
|  | 7 |  | -0.2 | B | 1.7 | A |  |
|  | 14 |  | -0.3 | B | - |  |  |
|  | 20 |  | -0.2 | B | - |  |  |
| CXCR3 | 3 | A | -0.7 | A | -0.5 | A | 0.018 |
|  | 7 |  | -0.6 | A | 0.5 | A |  |
|  | 14 |  | 0.6 | A | - |  |  |
|  | 20 |  | 0.0 | A | - |  |  |
